# Overview on Defocus Incorporated Multiple Segments Lenses: A Novel Perspective in Myopia Progression Management

**DOI:** 10.3390/vision6020020

**Published:** 2022-04-02

**Authors:** Matteo Mario Carlà, Francesco Boselli, Federico Giannuzzi, Gloria Gambini, Tomaso Caporossi, Umberto De Vico, Alfonso Savastano, Antonio Baldascino, Clara Rizzo, Raphael Kilian, Stanislao Rizzo

**Affiliations:** 1 Ophthalmology Department, Fondazione Policlinico Universitario A. Gemelli, IRCCS, 00168 Rome, Italy; francescoboselli@outlook.it (F.B.); federico.giannuzzi@gmail.com (F.G.); gambini.gloria@gmail.com (G.G.); tomaso.caporossi@gmail.com (T.C.); umbertodevico@gmail.com (U.D.V.); alfonso.savastano@policlinicogemelli.it (A.S.); antonio.baldascino@policlinicogemelli.it (A.B.); stanislao.rizzo@gmail.com (S.R.); 2 Ophthalmology Department, Catholic University “Sacro Cuore”, 00168 Rome, Italy; 3 Ophthalmology Unit, University of Verona, 37134 Verona, Italy; clararizzo2@gmail.com (C.R.); raphaelkilian8@yahoo.it (R.K.)

**Keywords:** myopia, myopia control, myopic defocus, spectacle lens, DIMS, defocus incorporated multiple segments, peripheral defocus, relative peripheral refraction

## Abstract

Myopia is becoming more common across the world, affecting approximately two billion people and rising. Different kinds of therapies (optical, pharmaceutical, environmental, or behavioral) have been proposed to decrease myopia progression, but with variable results and a lack of standardization. The evidence that targeted myopic defocus inhibits eye length growth has paved the way for several contact and spectacle lense designs to induce a peripheral defocus, thus slowing myopia progression, but the perfect configuration has yet to be defined. One of the newest and more promising approaches in this field is the use of Defocus Incorporated Multiple Segments (DIMS) lenses. These lenses are built from the assumption that targeted myopic defocus, produced by 396 mid-peripheral lenslets with positive power, inhibits eye length growth. Recent studies have highlighted the effectiveness of these lenses compared to children who had worn single vision spectacle lenses, in terms of myopia control and tolerability. Despite the evidence that these lenses can help slow down the progression of myopia, the occasional mid-peripheral aberrations they can induce, as well as the overall eye strain that comes with wearing them, should not be overlooked. The aim of this review is to give attention to the advantages and the shortfalls of this new approach and to evaluate its effectiveness in clinical practice.

## 1. Introduction

Myopia is becoming more common across the world, affecting approximately two billion people and still rising, expected to afflict half of the world’s population by 2050 [1,2,3,4]. The growing frequency of myopia in East Asian younger generations has made it an “epidemic” condition, especially in China, with a risk of reaching 95% prevalence in certain communities [5,6,7]. Higher myopia prevalence has also been seen in Europe, particularly among young adults [8,9]. As a result, myopia is now considered a substantial health-care burden [10]. In particular, the yearly incidence of myopia onset between the ages of 7 and 15 is steadily increasing, highlighting the need for standardized approaches in order to handle its development and avoid sight-threatening sequelae, such as myopic maculopathy, glaucoma, retinal detachment, and cataracts, conditions which may be lifelong [11].

A comprehensive treatment plan should be set up once a child has been diagnosed with myopia. In addition, several factors have to be considered, including the age of onset, baseline refractive status, visual environment, familiar compliance and parental myopia, risks and advantages of the eventual treatment, and yearly cost [4,12]. The most essential therapeutic aim, regardless of the treatment approach, is to decrease the course of myopia once it begins [1,5]. A variety of approaches have been explored, including pharmacological drops [13], environmental management [14], contrast-attenuation filters [15], or several kinds of optical manipulations [16,17,18].

## 2. Approaches for Myopia Management

Different kinds of therapies (optical, pharmaceutical, environmental, or behavioral) have been researched to prevent or postpone the beginning of myopia and to decrease its progression in order to minimize the associated ocular diseases connected to myopia. Regarding environmental approaches, several meta-analyses have shown that spending more time outside is associated with a lower incidence of myopia [18,19,20].

### 2.1. Pharmacological Treatment

Pharmacological treatment with low-dose 0.01% atropine has been shown to be the most effective strategy for decreasing myopic development in different studies, with successful rates varying from 45 to 77% regarding refractive error reduction, but no impact on axial length [4,18,21]. Although functionally successful, this approach displayed long-term adverse effects including photophobia, glare, and accommodation loss [18,22,23].

### 2.2. Ortho-Keratology

The research for alternatives has led to an increase in orthokeratology, or ortho-K (OK), which exploits specially designed and fitted contact lenses to temporarily reshape the cornea [24]. Orthokeratology has shown beneficial effects against myopia progression and axial elongation by 30 to 55 percent [25,26]. The most common complication of ortho-K treatment was corneal staining, with other clinically significant side effects including epithelial iron deposit, prominent fibrillary lines, and transient changes of corneal biomechanical properties, but no long-term effects on the corneal endothelium were observed [27]. In contrast, those contact lenses needed careful maintenance for eye health, because improper handling or cleaning increases the risk of infection, although the risk of microbial keratitis reported in a systematic review was found to be similar to other overnight corneal reshaping lenses [28,29].

### 2.3. Contact Lenses

Over time, bifocal (BFSCL) or multifocal soft contact lenses (MFSCL), peripheral gradient lenses, extended depth of focus (EDOF) contact lenses, and progressive addition lenses have all been explored to manage myopic development [16,30,31,32,33,34,35,36,37]. The first generation of bifocal or dual-focus lenses used a concentric zone of rings with plus power addition, resulting in a peripheral myopic defocus. These designs adopted a gradual increase in the positive power toward the periphery (progressive design) or featured discrete zones (concentric ring design), with the peripheral area of the lens having a considerably higher positive power. Concentric ring designs allow stronger axial elongation control than progressive ring designs, but refraction alterations are comparable [4]. Globally, these bifocal soft contact lenses reported a 30–38% reduction in myopia development regarding refraction, and a 31–51% reduction in axial length [38]. Moreover, other research studies have reported that the efficacy of bifocal contact lenses may improve in particular settings: increased usage time, high-rate myopic progression, structural designs with a greater hyperopic power in the mid-periphery [31]. MiSight is an example of a multizone design contact lens which has demonstrated decreased myopia progression (59%) and axial development of the eye (52%) during a three-year follow up [16,30].

Among these approaches, To Chi-ho and Carly Lam from the Centre for Myopia Research under PolyU’s School of Optometry have developed Defocus Incorporated Soft Contact (DISC) lenses [33], which consist of a central correction zone and a series of alternating defocusing and correction zones spreading to the perimeter in a 50:50 ratio, with the defocusing zones at +2.5 D, whereas the corrective zones match the distance prescription. As a result, the daily wearing of DISC lenses significantly slowed axial elongation and myopia progression by 25% when compared to controls [33]. A recent update in the contact lens approach for myopia control was reported by Walline et al. who conducted the BLINK clinical trial, in which children wearing high add power (+2.50 D) MFSCL had significantly less myopia progression and axial elongation over three years, when compared to SV lenses [39].

Similar to ortho-K, the contact lenses approach carries several management problems, linked with infections, corneal traumatism, and long-term biocompatibility.

### 2.4. Spectacle Lenses

In comparison to contact lenses and pharmaceutical therapies, intervention with spectacle lenses is a straightforward and less intrusive way for children and their parents, particularly for children under the age of eight [12]. The best prescription must be validated according to associated risk factors, taking into account several patient-specific characteristics connected to myopia development and progression [5]. Single-vision (SV) spectacle lenses have been found to provide less than a 14% decrease in myopia progression. In contrast to SV spectacle lenses, multiple articles have suggested that myopic defocus (MD) slows eye development and progression, whereas hyperopic defocus enhances eye growth [40,41,42]. Nevertheless, differently from previously thought, a recent systematic review reported that under-correction of myopia is not recommended as it did not slow myopia progression [43].

Many investigations have analyzed several kinds of new spectacle lens with relative peripheral defocus, with conflicting initial results [32,44]. Bifocals or progressive additional lenses (PALs) were introduced, allowing the user to see well at distances and up close, reducing accommodative strain and lag during prolonged near work [45]. In many investigations, these lenses showed therapeutic improvements ranging from 6% to 50% as compared to SV spectacles. A stronger impact was found in children with a higher degree of myopia (>3.0 D), accommodative lag, or near esophoria [45,46]. However, in comparison to single vision lenses (SVLs), bifocal spectacles or PALs had little impact in delaying myopia development during meta-analysis, with moderate-certainty evidence. Furthermore, the same meta-analysis highlighted that previously designed peripheral defocus-correcting lenses had mixed results in terms of refractive error and axial length, offering a low level of evidence regarding clinical results [47,48,49].

Since April 2021, a new variety of spectacle lenses with peripheral defocusing capability, known as Defocus Incorporated Multiple Segments (DIMS) technology, have been marketed from Hoya in Germany, Austria, and Switzerland under the trade name “MiYOSMART” (Hoya Lens Thailand Ltd., Bangkok, Thailand) [50]. This dual-focus spectacle lens, similarly to DISC contact lens, has a zonal structure with tiny, circular (≈1 mm diameter) lenslets in the mid-periphery, each with add power (+3.50 D). The novelty of this technology relies on the fact that images from each lenslet do not converge to generate a single image in the focal plane corresponding to the add power, but rather numerous distinct images. DIMS lenses have been reported to substantially delay myopia development when compared to single vision (SV) lenses in a recent clinical trial, while showing an absence of the negative effects of pharmaceutical therapies and a reduction in maintenance level when compared to contact lenses [50]. Several issues, however, remain to be investigated, including the quality of vision, which refers to the comfort and frequency of visual symptoms after wearing added-power spectral lenses and the efficacy of myopia control in long-term follow-ups [51].

Relying on the innovative results shown in recent articles, the aim of this review is to analyze the physical principle that forms the basis of DIMS lens construction and to assess its capacity to limit children’s myopia progression. Furthermore, given the possible disparities in the sensitivity to DIMS lenses in adults and children [52], along with the wide-ranging changes in developing eyes, this research aims to evaluate how such lenses may variably impact adults and adolescents, in terms of outcomes and tolerability.

## 3. DIMS Lens Structure

**Figure 1:**
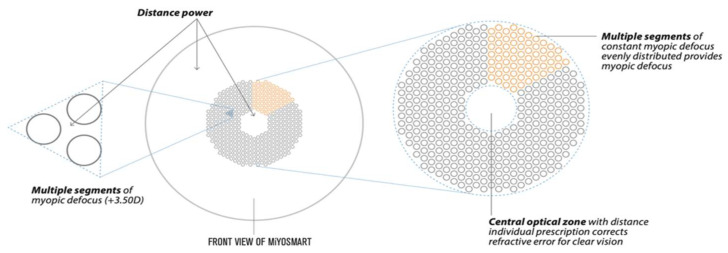
The design of the Defocus Incorporated Multiple Segments (DIMS) MiYOSMART spectacle lens. Available online: www.hoyavision.com (accessed on 30 December 2021).

The DIMS lens consists of polycarbonate with a refractive index of 1.590 and a spherical design, except for the mid-peripheral defocus area; Figure 1 shows a diagram of the optical design of a DIMS lens. DIMS lenses were provided by Hoya Co., Ltd. (Tokyo, Japan). The clear vision zone in the middle of the DIMS lens corresponds to the wearer’s central refractive power, and has a diameter of 9.4 mm. The mid-peripheral zone, also called the treatment zone, is made up of a honeycomb design area with a 50:50 area ratio between the clear vision area with the center refractive power (area between the unfilled circles) and the +3.50 diopters myopic defocus (unfilled circles), composed of 396 tiny lenslets, reaching a total of around 33 mm in diameter [52], as shown in Figure 1.

The maximum spheric power reachable by these lenses is −6.50, while for myopic astigmatism, the maximum value is −4.00 D; these maximum powers can be combined. The MiYOSMART lenses can also support a prismatic correction of up to 3.00 D per lens. Furthermore, this lens has a low-maintenance multi-coating which reduces the amount of light that reflects off lenses and is built to be water repellent and prevent water accumulation on the lens surface [53].

## 4. Optical Principles

In uncorrected myopia, the image shell lies centrally in front of the retina, while the peripheral wavefront partly lies behind the retina. Correction with a conventional spectacle lens shifts the focal plane of the optical image, causing it to lie centrally in the area of the fovea on the retina, but even more behind the retina in the periphery, causing a hyperopic peripheral defocus, which may encourage eye length expansion. This means that myopes usually have a hyperopic relative peripheral refraction (RPR), whereas emmetropes and hyperopes have a myopic RPR [54,55]. Previous research on the link between RPR and myopia onset has been contentious [56,57,58,59,60], with Hoogerheide et al. being the first to discover that, in their cohort, 65% of emmetropes and hyperopes who acquired myopia later had a hyperopic RPR [61]. A hyperopic RPR may be one of the indicators of myopia development, if assessed within two to four years before its onset, but its correlation with myopia progression was not well established, since RPR remained stable over the years after myopia onset, according to Mutti et al. [59].

In non-human primates, studies of the processes that govern refractive development have shown that hyperopic defocus may cause excessive eye growth and myopia. Targeted myopic defocus, solely in the retinal periphery, on the other hand, inhibits eye length growth. This also works when myopic defocus is offered in addition to the sharp retinal image in the macula [53,62]. Based on this assessment, simultaneous optics, named dual-focus optics, have been built, with concentric alternating powers in a zonal design inside the lens optic. This technique was reported to be effective in slowing eye development in a variety of animal models, including chickens, guinea pigs, and rhesus monkeys. When compared to control animals or fellow control eyes, there was a consensus among all researchers indicating that adding simultaneous myopic defocus to either hyperopic or plano correction might lower eye growth [63,64,65,66,67,68].

Human volunteers have been tested in clinical studies using this method of administering myopic defocus using a dual-focus optical device. Dual-focus soft contact lenses were developed, with the distance correction contained in a core zone big enough to provide acceptable visual acuity while still stimulating proper accommodation for close work, surrounded by concentric outer zones alternating the myopic defocus (extra positive power) and distance correction power. The zone sizes were chosen to ensure that myopic defocus was shown to the retina at all times [32]. As a result, the dual-focus lenses were reported to determine a lower mean axial elongation than the SV lenses.

Nevertheless, conflicting results have been presented regarding peripheral defocus spectacle lenses. Tarutta et al. reported the stabilization of refraction in myopic children wearing “perifocal-M” spectacle lenses, which determined the relative peripheral myopic defocus or reduced hyperopic defocus, when compared to SV lenses [69]. On the other side, when compared to SV lenses, Sankaridurg et al. showed no statistically significant differences in myopia retardation after using spectacle lenses with inbuilt myopic defocus in the periphery for a year [70].

**Figure 2:**
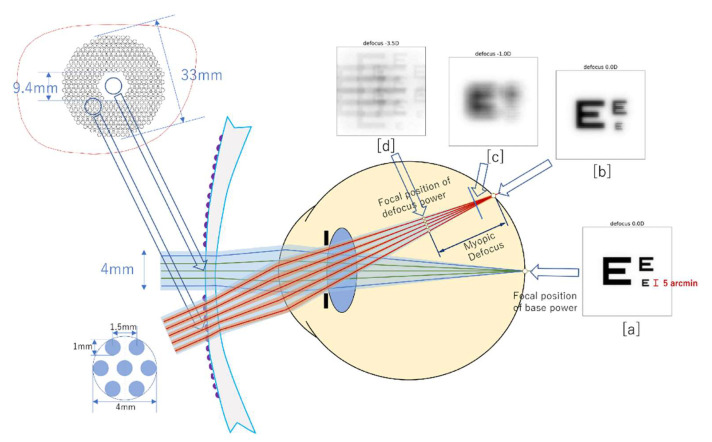
Optical principles of DIMS lens. The blue lines represent ray traces from the central (carrier) part of the lens, which form a clear image on the retina [**a**], while the red lines represent ray traces from the peripheral part of the lens, containing the lenslets, which form an image that is refracted by both the base part and the lenslets simultaneously [**b**]. The picture [**c**] or [**d**] will be generated on the retina if the target is close and the eye does not accommodate. An object located on the central axis generates a crisp image with no ghosting, while seeing a target via the peripheral section of the lens causes ghosting, depending on the relative refractive error at the retina as described in [**c**] or [**d**] [Image taken from Lam et al. “Effect of Defocus Incorporated Multiple Segments Spectacle LensWear on Visual Function in Myopic Chinese Children”] [71].

In DIMS lenses, the extra peripheral myopic defocus is produced by 396 tiny lenses inserted on the front surface of the single vision lens, in a ring-shaped region which surrounds a core correction zone, allowing for clear central vision and peripheral myopic defocus at the same time, [50,71] as seen in Figure 2. In addition to refractive correction, DIMS lenses may act on relative peripheral refraction (RPR). In fact, since this parameter has been employed to indirectly define retinal shape, changes in RPR following myopia management using myopic defocus lenses have been documented [72,73]. Jaskulski et al. tried to understand the effects of these lenses through aberrometric analysis, thanks to computer image-plane point-spread functions (PSF), modulation transfer functions (MTF), and simulated images [74]. The lenses, as expected, presented a peripheral defocus beam with a free 10-mm-wide central portion, without any help for the near target. According to the MTF analysis, the DIMS lens design can generate higher contrast images at low spatial frequencies than the traditional bifocal design, but they are unable to maintain this feature even with high spatial frequencies due to the fragmented aperture of the distance optic [74].

## 5. Prior Acknowledgements for DIMS Use

DIMS lens prescription for the children’s population requires a prior complete ocular examination. The first important step is an accurate measurement of the axial length (AL) along with cycloplegic autorefraction (because it can predict, in accordance with Tideman curves, the risk of acquiring high grade myopia [75]). In order to act from the earliest stages of disease progression, children with an age between 6 and 13 years old were the first to be targeted. A central spherical equivalent between −1.00 and −5.00 diopters with maximus astigmatism and anisometropia of 1.50 D and a monocular best corrected visual acuity of 0.00 logMAR were the most important including factors [50,51,71,76]. In contrast, strabismus and binocular vision abnormalities and prior experience of myopia control were the main exclusion factor [77].

The correct approach with DIMS glasses requires a minimum 15-h wear each day, underlining the importance of regularity in order to obtain the best result. These glasses must be carried with a perfectly suited centering, in order to assure a very good acuity in the central vision. Cases of a lack of the child’s acceptance could be attributed to an incorrect manner of wear: a very strict collaboration with an expert optician is fundamental for the good success of the therapy. In addition, in both children and the adult population, comprehensive information about the treatment compliance and efficacy is fundamental to optimize the effectiveness and tolerability of this device [53].

## 6. Outcomes on Myopia Progression

The first notable studies regarding the use of DIMS were published between 2019 and 2020. One of the main objectives of these studies was to assess the acceptability and adaptability of this type of lens. In particular, Lu et al. led a prospective cross-over study of 20 children who were recruited to wear both DIMS and SV lenses, randomly assigned [52]. The distance visual acuity (VA) in the primary gaze was tested under both standard (500 lux) and dim (50 lux) illumination, using high (100%) and low contrast (10%) visual charts (Early Treatment Diabetic Retinopathy Study charts, ETDRS), before and after 30 min of wearing DIMS or SV lenses. A VA approaching 40 cm across the mid-peripheral zone was also measured under these two levels of illumination [52]. They showed that, for DIMS lenses, near mid-peripheral VA was lowered by about 0.06 logarithms of the minimum unit angle of resolution in two of the four quadrants at standard illumination, and three quadrants at dim illumination, which indicates a small effect on mid-peripheral vision. In contrast, central VA was not affected by the DIMS lens compared to the SV lens under all circumstances [52].

In recent research, Lam and his colleagues released the first findings on the anti-myopic impact of DIMS lenses. They conducted a two-year double-masked randomized controlled study in which the starting population was composed of 183 Chinese children, aged 8–13 years with myopia between −1.00 and −5.00 D and astigmatism less than 1.50 D [50]. During this period, youngsters who wore DIMS were compared to children who wore single vision spectacle lenses, highlighting that children in the DIMS group grew 52% more slowly than those in the SV group regarding refractive progression (−0.85 D in the SV group vs. −0.41 D in the DIMS group). Similarly, children in the DIMS group had a 62% lower axial elongation than those in the SV group (mean difference 0.34 ± 0.04 mm among the two groups). Over the course of two years, 21.5% of the children who received DIMS lenses had no myopia progression, compared to 7.4% of the children who wore SV lenses [50].

Furthermore, the Lam study group continued this research to the third year of follow-up [76]. In this work, however, the cohort that previously wore spectacle lenses began wearing DIMS lenses, becoming the control-to-DIMS group, and a historical control group was added by reviewing clinical records. The study results claimed that only 5% and 2% of DIMS and control-to-DIMS participants, respectively, showed a myopia progression greater than 1.00 D, pointing out that axial elongation was less than 0.1 mm in 52% and 58% in the two groups, respectively [76]. Myopia progression and axial elongation in the DIMS group were comparable in the third year to those in the first and second years. On the other side, in the control-to-DIMS group, changes in the spherical equivalent refraction (SER) and AL in the third year were similar to the first-year changes in the DIMS group, even though these subjects were two years older. In fact, among those children, approximately 70% showed a progression of less than 0.25 D, suggesting that myopia control may be achieved even when starting to wear DIMS lenses at an older age. In both groups of DIMS users, the progression of myopia during the third year was significantly slower than in the historical control group (−0.35 D/0.18 mm of AL in the control group vs. −0.18 D/0.10 mm and −0.05 D/0.06 mm in the DIMS group and the control-to-DIMS group, respectively) [76]. All these results were consistent with those highlighted in the aforementioned three-year trial with dual-power contact lenses by Chamberlain [16] and the three-year trial with multifocal soft contact lenses by Walline [39] in reducing myopia progression.

A review of the results of different approaches for myopia management is visible in Table 1.

In addition to these findings, Zhang et al., referring to the same cohort of children, looked at changes in the relative peripheral refraction (RPR) in myopic children wearing DIMS and SV spectacle lenses, with the aim of evaluating variations in retinal shape following the use of myopic defocus [77]. The central and peripheral refractions across horizontal retinal eccentricities were determined in the straight-ahead position (center) and at 10°, 20°, and 30° at the nasal (10 N, 20 N, 30 N) and temporal (10 T, 20 T, 30 T) retinal eccentricities, respectively. The children demonstrated substantial changes in peripheral spherical equivalents at all retinal eccentricities after using DIMS lenses to cure myopia, indicating a consistent myopic shift along the horizontal retina. This could indicate that the DIMS group had a slower and more uniform eye development, while children in the SV group had a quicker axial expansion than that in the equatorial zone. Furthermore, the RPR changes differed across the two groups [77]. The SV group saw a considerable hyperopic RPR shift at the nasal retina, but no significant changes were shown in the temporal retina, indicating an asymmetric development. On the other side, the DIMS group showed a myopic RPR tendency in all sectors, although not statistically significant, except for a considerable difference for the RPR at 10 N when compared to the SV group, which suffered from a hyperopic shift in this sector. Since RPR might be utilized to define the retinal shape in an indirect manner, the higher hyperopic RPR seen in the SV group may indicate a steeper retinal shape, whereas the DIMS group was characterized by a flatter retinal shape [77].

## 7. Functional and Tolerability Outcomes

Successive studies have focused on understanding how the continuous wear of DIMS lenses could affect visual outcomes and binocular approach, including horizontal phoria, amplitude of accommodation, lag of accommodation, and stereopsis. In particular, major contributions are those made by the two-year results of Lam et al., who showed in a novel article that there were no statistically significant differences between the DIMS and SV groups when comparing changes in visual function from baseline to six-month visits [71]. Furthermore, children in both groups experienced a similar decrease in the monocular and binocular amplitude of accommodation and accommodative lag over the study period, even if these changes in accommodation were naturally linked with age increases in different studies [78,79,80]. Again, improvements in stereoacuity were similar among the two study groups, confirming that wearing DIMS lenses did not alter visual function measurements in a long-term follow-up, when compared to classical SV lenses [71].

The majority of studies have focused on refractive control and visual acuity, while tolerability outcomes such as ocular tiredness or straining have received much less attention. Asthenopia, headaches, and dizziness are all frequent adverse effects of increased refractive power in myopia or astigmatism prescriptions. Lu et al., in a recent study, made a comparison between the symptoms that SV lenses or DIMS lenses could cause. While the children’s group showed no statistical differences regarding complaints for eye strain, nausea, or dizziness, these problems were much more evident in the adults’ group [52]. After getting used to these lenses, both groups had no problems with tolerance, especially considering the possibility of myopia correction offered by this device [52].

In light of this, Ryu et al. compared two age cohorts to see whether DIMS lenses (as opposed to SV lenses) affect eye fatigue and visual performance in a difficult popular visual search paradigm (“Where’s Waldo”), in which a target figure was to be found in a highly-cluttered visual environment containing lots of distractor objects [81]. Participants reported a significant reduction in eye strain while wearing the DIMS lenses when compared to the lenses to which they were previously acclimated. This effect was detected in both the adolescents’ and the adults’ groups, demonstrating that DIMS lenses decreased the strain from the search task regardless of age [81]. One reason which could explain this reduced eye fatigue is linked to the optical arrangement of the DIMS lenses and the way they affect the visual processing of information and ongoing attentional processes. When compared to SV lenses, the quantity of information that needs to be processed peripherally is indeed reduced, since information outside the central focusing area is obscured to some extent, thus explaining a minor ocular effort [81].

## 8. DIMS Competitors

A recent report by Bao et al. analyzed newly-designed spectacle lenses with highly aspherical lenslets (HAL), named “Stellest” lenses (Essilor, Singapore), showing the interim one-year results in an ongoing two-year trial on Chinese children [82]. These lenses adopt a spherical front surface with 11 concentric rings made by contiguous aspherical lenslets aiming to provide a volume of myopic defocus, with distance correction supplied by the area of the lens without lenslets. The 54 children in the HAL group showed a significant slowdown in myopia progression when compared to the 52 children of the SVL group (mean changes in SER and AL to be −0.27 ± 0.06 D and 0.13 ± 0.02 mm vs. −0.81 ± 0.06 D and 0.36 ± 0.02 mm, respectively), with an overall 67% score in myopia control efficacy, similar to reported DIMS results [82]. Since this paper represents the first report of this novel device, further confirmation with the results obtained from the whole duration of the clinical trial are needed, with the possibility, in the future, to assess the differences in efficacy between DIMS and Stellest in a comparative study.

## 9. Conclusions

In recent years, increasing attention to the concept of peripheral defocus has led to the development of lenses that allow a slowdown in the progression of myopia, associated with good visual function. The recently marketed DIMS lenses have adopted myopic defocus of the mid-periphery to produce a significantly different peripheral refraction profile, in order to significantly control myopia development, most likely due to a change in total retinal shape.

When compared to SV spectacle lenses, daily use of the DIMS lens effectively delayed myopia progression and axial elongation in myopic kids after two years of follow up. The lenses provided clear visual acuity while simultaneously delivering MD to the eyes. When compared to pharmaceutical or contact lens therapies, this technique is simpler to administer and less invasive. Moreover, no significant differences between the visual function of the DIMS and SV were reported [50,52,76]. This result was confirmed even after three years of follow up, confirming DIMS’ role in myopia management. The best age to start therapy is yet unknown, and further research is needed to assess the treatment’s long-term effects. A three-year prospective, randomized, multicenter clinical trial in a larger myopic cohort of children treated with DIMS lenses is currently underway in China [51]. The findings, which will be available in a couple of years, will show whether and how different MD mechanisms may slow down myopia progression and axial lengthening.

An interesting innovation in this setting could be the evaluation of the possible role of pharmacologic therapy as enhancer or adjunct to the DIMS glasses. A combination of atropine and DIMS glasses could be used when the lenses are outside the tolerance range, in order to increase the therapeutic efficiency, but a standardized approach is still missing, opening possibilities for future lines of research.

In conclusion, DIMS lenses could be an important tool to reduce the progression of myopia by affecting the early stages of the development of the pathology, but long-term randomized studies are still needed to define their actual effectiveness, since their greater manageability and safety compared to contact lenses or drug therapy may promote their diffusion.

## Figures and Tables

**Table 1:** Clinical studies on myopia progression management. BF, bifocal spectacle lens; COMET2 and PEDIG, Correction of Myopia Evaluation Trial 2 Study Group and the Pediatric Eye Disease Investigator Group; DF, dual-focus contact lens; DISC, Defocus Incorporated Soft Contact; MD, myopic defocus; PAL, progressive addition lens; SV CL, single vision contact lens; SVL, single vision spectacle lens; DIMS, Defocus Incorporated Multiple Segments.

					Treatment Effect in Retarding Myopia Progression over the Study Period	
Study	Period (Months)	Age	Criteria of Rx (D)	Type of Interventions and Sample Size	Mean Difference in D (%)	Mean Difference in mm of AL (%)
Gwiazda et al. (2003) [45]	36	6–11	−1.25 to −4.5	SVL, *n* = 233; PAL (2 D Add), *n* = 229	−0.20 (14%)	−0.11 (15%)
Hasebe et al. (2008) [47]	18	6–12	−1.25 to −6	SVL, *n* = 44; PAL(1.5 D Add), *n* = 42	−0.31 (18%)	-
COMET2 and PEDIG (2011) [49]	36	8–12	−0.75 to −2.50	SV, *n* = 58 PAL (2 D Add), *n* = 52	−0.28 (24%)	-
Anstice and Phillips (2011) [32]	1st: 10 2nd: 20	11–14	−1.25 to −4.5	SV CL, *n* = 40 DF (2 D MD), *n* = 40	1st: −0.25 (37%) 2nd: −0.2 (54%)	1st: −0.11 (49%) 2nd: −0.12 (80%)
Sankaridurg et al., (2011) [35]	12	7–14	−0.75 to −3.5	SVL, *n* = 40 novel CL, *n* = 45	−0.29 (34%)	−0.13 (33%)
Lam et al. (2014) [33]	24	8–13	−1 to −5	SV CL, *n* = 63 DISC, *n* = 65	−0.21 (25%)	−0.11 (30%)
Chamberlain et al. (2019) [16]	36	8–12	−0.75 to −4	SV, *n* = 74 MiSight CL, *n* = 70	−0.73 (59%)	−0.32 (52%)
Walline et al. (2020) [39]	36	7–11	−0.75 to −5	SV, *n* = 98 High.add power CL, *n* = 98	−0.46 (43%)	−0.23 (36%)
Lam et al. (2020) [50]	24	8–13	−1 to −5	SV, *n* = 81 DIMS, *n* = 79	−0.55 (52%)	−0.32 (62%)
Lam et al. (2021) [76]	12 (3rd year of previous trial)	10–15	−1 to −5	Control group, *n* = 76 Control-to-DIMS, *n* = 55	−0.30 (86%)	−0.12 (61%)

## Data Availability

The data that support the findings of this study are available from the corresponding author (M.M.C.) upon reasonable request.
